# Free fatty acids support oligodendrocyte survival in a mouse model of amyotrophic lateral sclerosis

**DOI:** 10.3389/fncel.2023.1081190

**Published:** 2023-05-12

**Authors:** Takashi Maruyama, Shogo Tanabe, Akiko Uyeda, Tatsunori Suzuki, Rieko Muramatsu

**Affiliations:** ^1^Department of Molecular Pharmacology, National Institute of Neuroscience, National Center of Neurology and Psychiatry, Tokyo, Japan; ^2^Department of Pharmacoscience, Graduate School of Pharmaceutical Sciences, Tokyo University of Science, Chiba, Japan; ^3^Department of Pharmaceutical Sciences, Graduate School of Pharmaceutical Sciences, Tokyo University of Science, Chiba, Japan

**Keywords:** free fatty acids, amyotrophic lateral sclerosis (ALS), oligodendrocyte, lipidome, SOD1

## Abstract

**Introduction:**

Amyotrophic lateral sclerosis (ALS) is a fatal neurodegenerative disease characterized by the white matter degeneration. Although changes in blood lipids are involved in the pathogenesis of neurological diseases, the pathological role of blood lipids in ALS remains unclear.

**Methods and results:**

We performed lipidome analysis on the plasma of ALS model mice, mutant superoxide dismutase 1 (SOD1^*G*93*A*^) mice, and found that the concentration of free fatty acids (FFAs), including oleic acid (OA) and linoleic acid (LA), decreased prior to disease onset. An *in vitro* study revealed that OA and LA directly inhibited glutamate-induced oligodendrocytes cell death via free fatty acid receptor 1 (FFAR1). A cocktail containing OA/LA suppressed oligodendrocyte cell death in the spinal cord of SOD1^*G*93*A*^ mice.

**Discussion:**

These results suggested that the reduction of FFAs in the plasma is a pathogenic biomarker for ALS in the early stages, and supplying a deficiency in FFAs is a potential therapeutic approach for ALS by preventing oligodendrocyte cell death.

## 1. Introduction

Amyotrophic lateral sclerosis (ALS) is a progressive and fatal degenerative disease primarily characterized by selective loss of upper and lower motor neurons (MNs), muscle wasting, and paralysis (Brown and Al-Chalabi, 2017). Approximately 90% of ALS cases are sporadic, and the remaining 10% are inherited with mutations in genes such as superoxide dismutase 1 (SOD1). Because of the genetic diversity and heterogeneous disease progression, it takes approximately 1 year to diagnose ALS from the first symptom (Cellura et al., 2012). As early intervention is a promising therapeutic approach for neurodegenerative diseases, biomarkers that can facilitate early diagnosis and improve the prognosis of ALS are urgently needed (Sturmey and Malaspina, 2022).

Increased energy expenditure, hypermetabolism, and alterations in several metabolites, including lipids, have been reported in patients with both sporadic and familial ALS, as well as in rodent models (Dupuis et al., 2004; Godoy-Corchuelo et al., 2022). Lipids comprise diverse groups of molecules and act not only as energy sources but also as components of the cell membrane and signaling molecules that regulate a variety of cellular responses via receptors or transporters expressed on the cell surface in various organs, including the central nervous system (CNS) (Cermenati et al., 2015). Disruptions in the lipid pathways within the CNS have been implicated in triggering neurological pathologies in ALS (Chaves-Filho et al., 2019; Tracey et al., 2021). Moreover, the importance of systemic lipid homeostasis in ALS pathology is suggested by the fact that increased dietary lipids provide neuroprotective effects and extend survival (Dupuis et al., 2004; Mattson et al., 2007), whereas restricted calorie intake aggravates neurological symptoms in ALS model mice (Pedersen and Mattson, 1999). However, global changes in systemic lipid metabolites at the early stages of ALS progression and their links to CNS pathogenesis remain unclear.

Emerging evidence has suggested that ALS is not merely a disease of MNs, and that their interactions with glial cells, including astrocytes, microglia, and oligodendrocytes (OLs), also mediate the pathology of ALS (Boillée et al., 2006; Yamanaka et al., 2008; Puentes et al., 2016). Compared to other CNS glial cells, OLs have distinct physiological functions, including forming a myelin sheath to ensure neuronal axon integrity, rapid conduction, and providing metabolic support for neurons (Lee et al., 2012; Nave and Werner, 2014). OL dysfunctions and demyelination have been reported in ALS patients (Kang et al., 2013). In a rodent ALS model (SOD1^*G*93*A*^ mice), degeneration of mature OLs and increased proliferation of oligodendrocyte precursor cells were observed prior to the appearance of neurological symptoms (Kang et al., 2013; Philips et al., 2013), suggesting that OLs mediate the early pathogenesis of ALS. We and others have reported that peripheral-derived factors, including hormones and immune cells, influence the cellular response of OLs and oligodendrocyte precursor cells in CNS disease, as vascular damage often occurs in the lesion (Kuroda et al., 2017; Hamaguchi et al., 2019; Saitoh et al., 2022). Moreover, OLs require lipids for development and function (Montani, 2021). Thus, we hypothesized that alterations in circulating lipids in ALS may affect oligodendrocyte function, thereby mediating the early pathogenesis of ALS.

In this study, we conducted a non-targeted lipidomic analysis of circulating lipids in the plasma of SOD1^*G*93*A*^ mice and found a robust decrease in subsets of FFAs, including oleic acid (OA) and linoleic acid (LA), before the onset of symptoms. In primary cultured murine oligodendrocytes, OA/LA inhibited excitotoxic oligodendrocyte death through FFAR1. Systemic LA/OA administration before disease onset ameliorated OL and MN deaths in SOD1^*G*93*A*^ mice.

## 2. Materials and methods

### 2.1. Ethics

All experimental procedures were approved by the Committee on the Ethics of Animal Experiments of the National Institutes of Neuroscience, National Center of Neurology and Psychiatry (2021013R2).

### 2.2. Mice

Postnatal day 1 (P1) C57BL/6J mice were obtained from Tokyo Laboratory Animals Science. SOD1-G93A transgenic mice, that is express a G93A mutant form of human SOD1 were obtained from Jackson Laboratory (#002726) and heterozygous (SOD1^*G*93*A*^) males were bred with wild-type (WT) female C57BL/6J mice (Japan SLC). Offspring were ear punched and genotyped using PCR with following primers: *Human/Mouse Sod1* forward, CAGCAGTCACATTGCCCARGTCTCCAACATG; *Human Sod1* reverse, CCAAGATGCTTAACTCTTGTAATCAATGGC; *Mouse Sod1* reverse, GTTACATATAGGGGTTTACTTCATAATCTG. Mice not expressing the transgene were used as WT littermate controls. Mice were housed in an air-conditioned room at 22°C with a 12-h light–dark cycle, had free access to water and food, and were maintained in sterile, pathogen-free conditions. The mice were fed standard chow diets (CE-2, CLEA Japan) and water under *ad libitum* conditions.

Female SOD1^*G*93*A*^ mice were intraperitoneally administered with linoleic acid-oleic acid-albumin (10.6 mg/kg, L9655, Sigma-Aldrich) or bovine serum albumin (BSA; 1.25 g/kg, 810017, Sigma-Aldrich) twice a week between P60 and P100.

### 2.3. Plasma preparation and lipidomics

Cardiac blood was collected via cardiac puncture from P60, P100 SOD1^*G*93*A*^, or WT mice under anesthesia, mixed with one-hundredth of 1.3% ethylenediaminetetraacetic acid-2K/0.9% saline, and centrifuged at 1,200 rpm for 15 min at 4°C. The supernatant was collected as plasma and stored at -80°C until using. Untargeted and unbiased lipidomic analysis were conducted at Human Metabolome Technologies Inc. (HMT, Tsuruoka, Japan).

### 2.4. Primary culture of oligodendrocytes

Primary cultures of oligodendrocytes were obtained from mice at P1 as previously described (Hamaguchi et al., 2019). The forebrains were dissected and minced with fine scissors in ice-cold phosphate-buffered saline (PBS). The minced tissues were dissociated with 0.25% trypsin (15090–046, Thermo Fisher Scientific, Waltham, MA, USA) in PBS at 37°C for 10 min. After neutralization with Dulbecco’s modified Eagle’s medium (DMEM; 12800082, Thermo Fisher Scientific) containing 10% fetal bovine serum (FBS; F7524, Sigma-Aldrich), cells were centrifuged at 1,500 rpm for 10 min, resuspended in 10% FBS-DMEM, and filtered through a 70 μm nylon cell strainer. Cells were then plated on poly-L-lysine (PLL; P2636, Sigma-Aldrich)-coated 10-cm dishes at a density of 5 × 10^5^ cells/dish and maintained at 37°C with 5% CO_2_ in 10% FBS-DMEM. 10 days after culturing, cells were washed with PBS and the remaining cells were treated with 0.05% Trypsin-PBS at 37°C for 3 min. The detached cells were filtered through a 40 μm nylon cell strainer and plated into non-coated dishes and incubated at 37°C for 30 min. Then, the non-adherent cells were collected and plated into PLL-coated 96 well glass-bottom plate (5866-960, IWAKI) at a density of 5 × 10^4^ cells/well in culture medium, consisting of DMEM/Nutrient Mixture F-12 Ham medium (DMEM/F12; D0547, Sigma-Aldrich, St. Louis, MO, USA) supplemented with 1 mM sodium pyruvate (S8636, Sigma-Aldrich, St. Louis, MO, USA), 0.1% bovine serum albumin (BSA; 810017, Sigma-Aldrich, St. Louis, MO, USA), 50 μg/ml apo-transferrin (T5391, Sigma-Aldrich), 5 μg/ml insulin (I1882, Sigma-Aldrich), 30 nM sodium selenite (S9133, Sigma-Aldrich), 10 nM biotin (B4639, Sigma-Aldrich), 10 nM hydrocortisone (H6909, Sigma-Aldrich), 10 ng/ml platelet-derived growth factor-AA (315-17, PeproTech), and 10 ng/ml basic fibroblast growth factor (450-33, PeproTech). For the purification of oligodendrocytes progenitor cells (OPCs), detached cells were treated with CD140a (PDGFRα) Microbead kit (130-101-547, Miltenyi Biotec, Bergisch Gladbach, Germany) and the OPCs were plated onto PLL-coated 96-well glass plate at a density of 5.0 × 10^4^/well in culture medium. We confirmed that 90.26 ± 0.93% of cells in the culture were labeled by Olig2, an oligodendrocyte lineage cell marker, using immunocytochemical analysis (data not shown).

Three days after culturing, mouse siGENOME siRNAs (Horizon Discovery) for target genes were transfected into cultured oligodendrocytes using Lipofectamine RNAiMAX (13778075, Thermo Fisher Scientific, Waltham, MA, USA). Four hours after transfection, the cells were treated with differentiation medium and cultured for additional 3 days. Differentiation medium consisted of DMEM/F12 containing 1 mM sodium pyruvate, 0.1% BSA, 50 μg/ml apo-transferrin, 5 μg/ml insulin, 30 nM sodium selenite, 10 nM biotin, 10 nM hydrocortisone, and 20 ng/ml triiodo-L-thyronine (T2752, Sigma-Aldrich). Then the cells were treated with 30 μM Linoleic Acid-Oleic Acid-Albumin (L9655, Sigma-Aldrich) (Ahn et al., 2013; Lee et al., 2022) and 100 μM glutamate (G5889, Sigma-Aldrich, St. Louis, MO, USA) for 24 h, and dead cells were stained with 1 μg/mL Propidium Iodide (PI; 169-26281, WAKO) for 30 min.

### 2.5. Immunocytochemistry

Cells were fixed with 4% paraformaldehyde (PFA, Merck, Darmstadt, Germany) in PBS at room temperature for 30 min, followed by permeabilization and blocking with blocking solution (0.1% Triton X-100 and 3% BSA in PBS) for 1 h at room temperature. Then, the cells were incubated with the primary antibody, rat anti-myelin basic protein (MBP) antibody (AB7349, Abcam, Cambridge, UK) at 1:500, and rabbit anti-Cleaved caspase-3 (CC3; #9661, Cell Signaling Technology, Danvers, MA, USA) at 1:1000 dilution in the blocking solution overnight at 4°C. Primary antibody was detected by the Alexa Fluor 488-conjugated donkey antibody against rat IgG (Thermo Fisher Scientific) diluted with blocking solution at 1:500. Nuclei were stained with 4′,6-diamidino-2-phenylindole (DAPI, 1 mg/ml, Dojindo Laboratories, Kumamoto, Japan). Images were acquired using a confocal laser-scanning microscopy (FV3000, Olympus, Tokyo, Japan) with a 20×/0.75 objective lens. To estimate oligodendroglial cell death, the percentage of PI^+^ MBP^+^ cells in MBP^+^ cells were calculated from more than 50 MBP^+^ cells using ImageJ software (National institute of health).

### 2.6. Quantitative reverse transcription polymerase chain reaction (qRT-PCR)

Three days after siRNAs transfection, total RNA was isolated from cultured oligodendrocytes using the TRIzol reagent (10296010, Thermo Fisher Scientific, Waltham, MA, USA). For quantitative RT-PCR, cDNA was synthesized using the High-Capacity cDNA Reverse Transcriptase Kit (4368814, Applied Biosystems, Waltham, MA, USA). Real-time qRT-PCR was performed using KAPA SYBR Fast Master Mix (7959397001, KAPA Biosystems, Wilmington, MA, USA) with the following primer pairs: *Ffar1* forward, GGGCTTTCCATTGAACTTGTTAG; *Ffar1* reverse, GCCCAGATGGAGAGTGTAGACC; *Gapdh* forward, AGGTCGGTGTGAACGGATTTG; *Gapdh* reverse, TGTAGACCATGTAGTTGAGGTCA. PCR conditions included one cycle at 95°C for 30 s, followed by 39 cycles of 95°C for 5 s and 60°C for 45 s. A melting analysis was carried out following PCR to monitor amplification specificity. Relative mRNA expression was normalized against *Gapdh* mRNA levels in the same samples and calculated by the Δ/Δ-Ct method.

### 2.7. Immunohistochemistry

Mice were transcardially perfused with 4% PFA in PBS. Lumbar spinal cords were post-fixed with 4% PFA in PBS overnight at 4°C and then immersed in 30% sucrose in PBS at 4°C. Tissues were embedded in optimal cutting temperature compound (Tissue-Tek, Sakura Finetek), sliced into 30 μm sections and mounted on Adhesive Glass Slides (Matsunami Glass). Sections were permeabilized with 0.1% Triton X-100 in PBS and blocked with 3% normal donkey serum in PBS for 1 h at room temperature. The sections were incubated with primary antibodies overnight at 4°C and then incubated with fluorescently labeled secondary antibodies for 1 h at room temperature. The following primary antibodies were used: rabbit anti-CC3 (#9661, Cell Signaling Technology, Danvers, MA, USA, 1:1000), goat anti-Olig2 (AF2418, R&D Systems, 1:1000), mouse anti-APC (CC1; OP80, Calbiochem, 1:1000), rabbit anti-Iba1 (019-19741, Wako, 1:1000), mouse anti-glial fibrillary acidic protein (GFAP; G3893, Sigma-Aldrich, St. Louis, MO, USA, 1:1000), goat anti-choline acetyltransferase (ChAT; AB144P, Millipore, 1:1000), rabbit anti-GPCR GPR40 (FFAR1; ab236285, Abcam, Cambridge, UK, 1:500) antibodies. Alexa Fluor 488-conjugated donkey antibodies against rabbit, or mouse IgG, Alexa Fluor 568-conjugated donkey antibodies against mouse or goat IgG, and Alexa Fluor 647-conjugated donkey antibody against rabbit IgG (Thermo Fisher Scientific, Waltham, MA, USA) were used as secondary antibodies. Images were acquired using a confocal laser-scanning microscopy (FV3000, Olympus, Tokyo, Japan) with a 20×/0.75 objective lens. Oligodendroglial cell death were evaluated with percentage of CC3^+^ CC1^+^ Olig2^+^ cells in CC1^+^ Olig2^+^cells and neuronal cell death were evaluated with the number of ChAT^+^ cells in the anterior horn using ImageJ. To evaluate the gliosis, fluorescence intensity of GFAP and Iba1 in the anterior horn was normalized to its area using ImageJ.

### 2.8. Grip strength test

The grip strength test for OA/LA or BSA-treated SOD1^*G*93*A*^ mice were performed twice a week between P60 and P100. The mice were placed on a grid attached to grip strength meter (Bio-GS3; Bioseb). The tail was pulled until the mouse released the grid, and the maximum value (given by gram) from 10 trials was recorded.

### 2.9. Statical analysis

All statistical values are presented as mean values ± standard error of mean (SEM). The number of samples analyzed is given for each experiment. Significant differences were determined with Student’s *t*-test, one-way analysis of variance (ANOVA) followed by Tukey–Kramer test, or two-way ANOVA followed by Sidak’s multiple comparison tests. All data were analyzed using Excel 2019 (Microsoft) or EZR (Kanda, 2013).

## 3. Results

### 3.1. Reduced circulating free fatty acid in SOD1^*G*93*A*^

**FIGURE 1:**
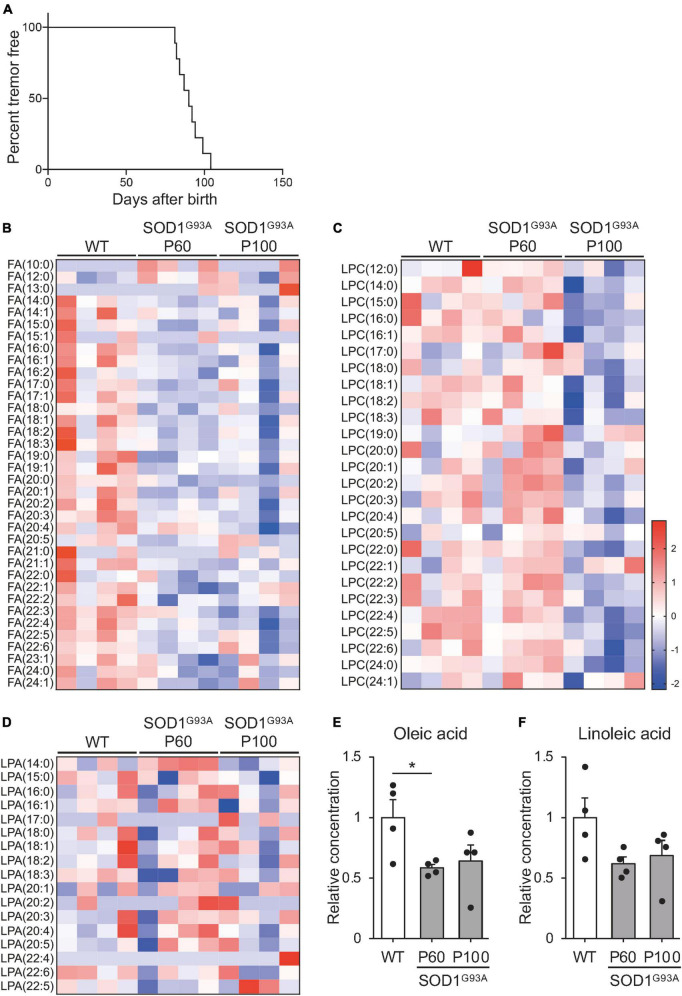
Free fatty acid (FFA) concentration is reduced in the plasma of SOD1^G93A^ mice. The plasma was obtained from WT or SOD1^G93A^ mice at P60 and P100, and subjected to mass spectrometry for lipid analysis. **(A)** Kaplan–Meier curve showing the probability of SOD1^G93A^ mice without tremor symptoms (*n* = 9). **(B–D)** A heat map showing the z-score of each fatty acid **(B)**, lysophosphatidylcholine **(C)**, and lysophosphatidic acid **(D)** in the plasma. The rows and columns represent lipids and samples, respectively **(E,F)**. Graph indicating the relative concentration of oleic acid (OA) **(E)**, and linoleic acid (LA) **(F)** in the plasma. The error bars represent mean ± SEM (*n* = 4 for each group). *P < 0.05, assessed by one-way ANOVA followed by Tukey–Kramer test for panel **(E)**.

There are few reports on changes in blood lipid levels before the appearance of symptoms. To investigate how circulating lipids are altered with ALS progression, we performed global lipidomic analysis of plasma from SOD1^*G*93*A*^ mice at the pre-symptomatic (P60) and symptomatic (P100) stages (Weydt et al., 2003; Kaiser et al., 2006; Rudnick et al., 2017). Consistent with previous reports, no SOD1^*G*93*A*^ mice exhibited hindlimb tremor symptoms at P60, while 88.8% of SOD1^*G*93*A*^ mice showed symptoms at P100 (Figure 1A). When compared to WT (P100) controls, SOD1^*G*93*A*^ mice exhibited a decrease in subsets of FFAs from the pre-symptomatic stage, which was sustained during the progression of pathology (Figure 1B and Supplementary Table 1). Conversely, we found a decrease in lysophosphatidylcholine from the symptomatic stage (Figure 1C) and no significant changes in lysophosphatidic acid (Figure 1D). Thus, we decided to focus on FFA during ALS progression. FA (16:0), FA (16:1), FA (17:0), FA (17:1), FA (18:0), FA (18:1), FA (19:0), FA (19.1), FA (20:0), FA (20:1), FA (20:2), FA (20:3), FA (21:1), FA (22:1), FA (22:3), FA (22:4), FA (22:5), and FA (22:6) were significantly reduced in SOD1^*G*93*A*^ mice at P60 (Supplementary Table 1). Among the downregulated FFA species, OA (FA 18:1), a monosaturated 18-carbon fatty acid that is one of the most common types of fatty acid in nature, was relatively abundant in circulation (Gonçalves-de-Albuquerque et al., 2012) and showed a significant decrease before the onset (Figure 1E and Supplementary Table 1, 58.5 ± 3.0% at P60 compared to WT, *P* = 0.0312). Among other 18-carbon fatty acids, LA (FA 18:2), a polyunsaturated fatty acid, also showed the second highest level in physiological conditions (Supplementary Table 1), and a similar decreasing trend was observed in the early stage of ALS pathogenesis (Figure 1F, 62.0 ± 5.6% at P60 compared to WT, *P* = 0.075). Notably, OA and LA have been implicated to facilitate neuroprotective effects in neurodegenerative diseases (Vesga-Jiménez et al., 2022). In addition, the mixture of OA and LA has been shown to exert synergistic effect on various cellular processes (Belal et al., 2018). These facts prompted us to investigate whether OA and LA are involved in ALS pathogenesis.

### 3.2. Oleic and linoleic acid prevent oligodendroglial cell death through Ffar1

**FIGURE 2:**
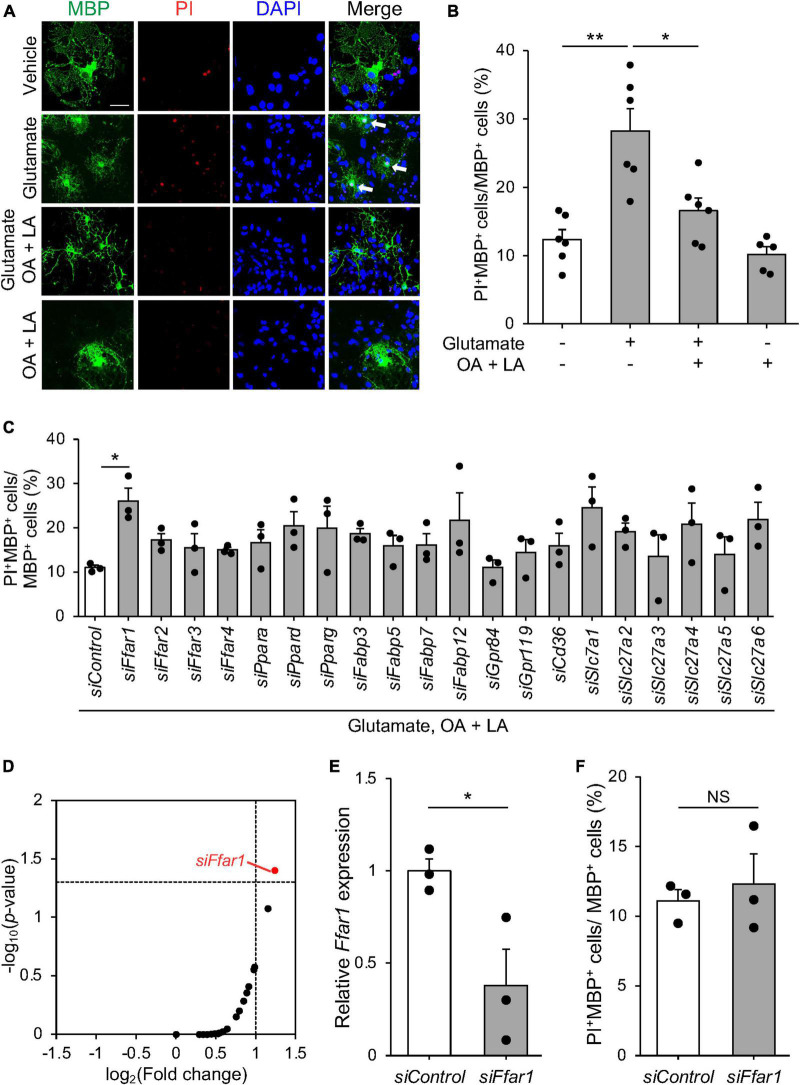
FFAR1 is a candidate molecule that regulates oligodendrocyte cell death. **(A)** Representative images show the fluorescence of PI (red) and immunocytochemistry for MBP (green) of primary oligodendrocytes treated with vehicle, glutamate, glutamate + OA + LA, OA + LA. White arrows indicate PI^+^ MBP^+^ cells. Scale bar: 30 μm. **(B)** Graph indicating the percentage of PI^+^ MBP^+^ cells in MBP^+^ cells (vehicle: *n* = 6, glutamate: *n* = 6, glutamate + OA + LA: *n* = 6, OA + LA: *n* = 5). **(C)** Graph showing the percentage of PI^+^ MBP^+^ cells in MBP^+^ cells of primary oligodendrocyte culture transfected with siRNA library followed by treatment with glutamate + OA + LA (*n* = 3 for each). **(D)** Plot indicating the –log_10_ (*p*-value) and log_2_ (fold change) of PI^+^ MBP^+^ cell percentage compared with control siRNA in each siRNA treatment (*n* = 3 for each group). **(E)** Relative expression of *Ffar1* mRNA in primary oligodendrocytes after control or *Ffar1* siRNA treatment (*n* = 3 for each group). **(F)** The percentage of PI^+^ MBP^+^ cells in MBP^+^ cells after *Control* or *Ffar1* siRNA treatment (*n* = 3 for each group). Error bars represent mean ± SEM, **P < 0.01, *P < 0.05, assessed by one-way ANOVA followed by Tukey–Kramer test for panels **(B–D)**, and by two-sided Student’s *t*-test for panels **(E,F)**, NS, not significant.

Oligodendrocyte death, including apoptosis, has been reported in pre-symptomatic SOD1^*G*93*A*^ mice (Kang et al., 2013; Philips et al., 2013; Ferraiuolo et al., 2016). Excitotoxic damage has been implicated in oligodendrocyte death in CNS diseases associated with OL degeneration (Matute et al., 2007; Foran and Trotti, 2009). FFAs, including OA and LA have been suggested to inhibit apoptosis via G protein-coupled receptors (Katsuma et al., 2005). This prompted us to investigate whether FFAs support oligodendrocyte survival by inhibiting apoptosis. To test this hypothesis, we examined whether OA and LA prevented oligodendrocyte death induced by glutamate-induced excitotoxicity in murine primary cultured OLs. Treatment with glutamate increased PI^+^ dead cells in MBP^+^ oligodendrocytes, consistent with previous reports (Oka et al., 1993; Matute et al., 2006), while an increase in the population of PI^+^ oligodendrocytes was diminished in the presence of the OA/LA cocktail (Figures 2A, B). OA/LA alone did not affect the death of OLs (Figures 2A, B). These results suggested that OA/LA prevent excitotoxicity-induced OL death.

Free fatty acids exert various cellular responses through receptors and transporters expressed in cells. To elucidate the molecular mechanisms by which FFAs regulate oligodendrocyte survival, we conducted siRNA-based functional screening for known cell surface receptors (FFAR1-4, GPR84, and 119) (Kimura et al., 2020), intercellular transporters (CD36 and SLC27a1-6) (Kazantzis and Stahl, 2012), intracellular transporters (FABP3, 5, 7, and 12) (Falomir-Lockhart et al., 2019), and nuclear receptors (PPARα, δ, and γ) (Varga et al., 2011) for FFAs. We used a commercially available siRNA library consisting of siRNA pools that include four distinct siRNAs targeting different regions of genes (Mukherjee et al., 2020; Allan et al., 2021; Simoneschi et al., 2021; Uyeda et al., 2021). Among the tested genes, only siRNA targeting *Ffar1* exhibited a significant reduction in the anti-cell death effect of OA/LA (Figures 2C, D). RT-PCR analysis confirmed the inhibition of *Ffar1* expression (Figure 2E), whereas *Ffar1* knockdown itself did not affect oligodendrocyte survival (Figure 2F). These results suggest that OA/LA prevents excitotoxic cell death in oligodendrocytes via FFAR1.

**FIGURE 3:**
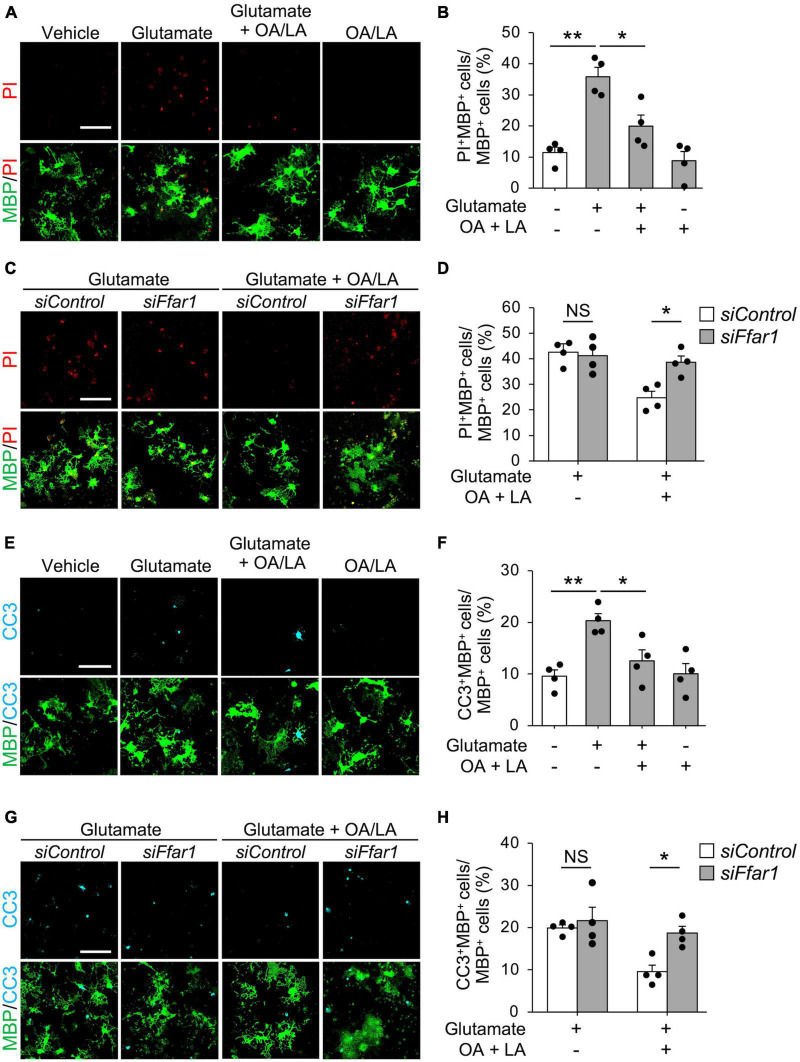
OA and LA inhibit glutamate-induced oligodendrocyte apoptosis via FFAR1. **(A)** Representative images show the fluorescence of PI (red) and immunocytochemistry for MBP (green) of purified primary oligodendrocytes treated with vehicle, glutamate, glutamate + OA/LA, and OA/LA. Scale bar: 100 μm. **(B)** Graph showing the percentage of PI^+^ MBP^+^ cells in MBP^+^ cells assessed from panel **(A)** (Vehicle: *n* = 4, glutamate: *n* = 4, glutamate + OA/LA: *n* = 4, and OA/LA: *n* = 4). **(C)** Representative images showing the fluorescence of PI (red) and immunocytochemistry for MBP (green) in purified primary oligodendrocytes treated with *control* siRNA or *Ffar1* siRNA in the presence of glutamate with or without OA/LA. Scale bar: 100 μm. **(D)** Graph showing the percentage of PI^+^ MBP^+^ cells in MBP^+^ cells (*Control* siRNA in glutamate: *n* = 4, *Control* siRNA in glutamate + OA/LA: *n* = 4, *Ffar1* siRNA in glutamate + OA/LA: *n* = 4, and *Ffar1* siRNA in glutamate + OA/LA: *n* = 4). **(E)** Representative images showing fluorescence of immunocytochemistry for CC3 (cyan) and MBP (green) of purified primary oligodendrocytes treated with vehicle, glutamate, glutamate + OA/LA, and OA/LA. Scale bar: 100 μm. **(F)** Graph showing the percentage of CC3^+^ MBP^+^ cells in MBP^+^ cells assessed from panel **(E)** (vehicle: *n* = 4, glutamate: *n* = 4, glutamate + OA/LA: *n* = 4, and OA/LA: *n* = 4). **(G)** Representative images showing the fluorescence of immunocytochemistry for MBP (green) and CC3 (cyan) of purified primary oligodendrocytes treated with *control* siRNA or *Ffar1* siRNA in the presence of glutamate with or without OA/LA. Scale bar: 100 μm. **(H)** Graph showing the percentage of CC3^+^ MBP^+^ cells in MBP^+^ cells assessed from panel **(G)** (*Control* siRNA in glutamate: *n* = 4, *Control* siRNA in glutamate + OA/LA: *n* = 4, *Ffar1* siRNA in glutamate + OA/LA: *n* = 4, and *Ffar1* siRNA in glutamate + OA/LA: *n* = 4). NS, not significant, *P < 0.05, **P < 0.01, assessed by one-way ANOVA followed by Tukey–Kramer test for panels **(B,F)**, and two-way ANOVA followed by Sidak’s multiple comparison tests for panels **(D,H)**.

Oleic acid/linoleic acid also affect astrocytes (Murphy, 1995; Nakajima et al., 2019), which were present in the culture we used in our study. Thus, we further investigated whether OA/LA directly facilitated anti-cell death effects on oligodendrocytes through FFAR1 by purification culture of PDGFRα^+^ OPCs using magnetic-activated cell sorting (MACS), which allows high-purity culture of Olig2^+^ cells (90.26 ± 0.93%). We found that OA/LA suppressed glutamate-induced oligodendrocyte cell death (Figures 3A, B), and silencing *Ffar1* expression significantly inhibited anti-cell death effect of OA/LA in purified oligodendrocyte culture (Figures 3C, D), indicating that OA/LA directly supported oligodendrocyte survival via FFAR1. To investigate whether OA/LA inhibited apoptosis, we analyzed cleaved-caspase3^+^ (CC3, an apoptosis marker) in purified oligodendrocytes culture treated with glutamate in the presence of OA/LA. The result revealed that treatment with glutamate increased CC3^+^ MBP^+^ oligodendrocytes, while an increase in the population of CC3^+^ oligodendrocytes was diminished in the presence of the OA/LA cocktail (Figures 3E, F). Furthermore, inhibition of *Ffar1* expression significantly diminished the anti-apoptotic effect of OA/LA (Figures 3G, H), indicating that OA/LA directly supported oligodendrocyte survival via FFAR1.

### 3.3. OL/LA supports oligodendroglial survival in SOD1^*G*93*A*^

**FIGURE 4:**
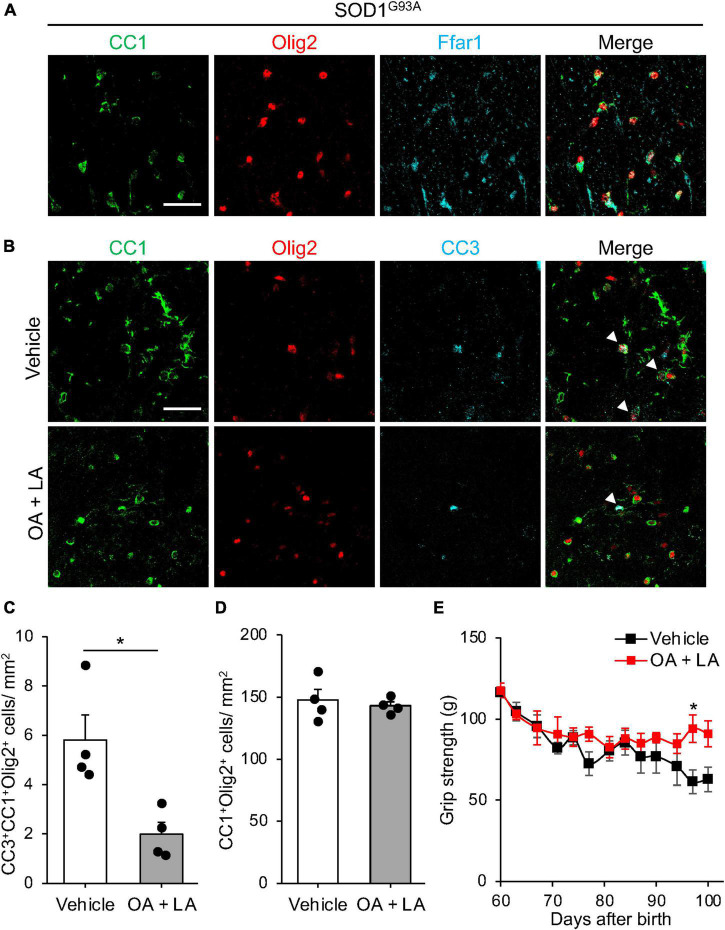
OA and LA prevent the OL loss in SOD1^G93A^ mice. **(A)** Representative images show the immunohistochemistry for CC1 (green), Olig2 (red), and Ffar1 (cyan) in the spinal cord section of SOD1^G93A^ at P100. **(B)** Representative images indicate immunohistochemistry for CC1 (green), Olig2 (red), and CC3 (cyan) in the spinal cord of Vehicle or OA + LA injected SOD1^G93A^ mice at P100. White arrows indicate CC1^+^ Olig2^+^ CC3^+^ cells. **(C)** Graph shows the number of CC1^+^ Olig2^+^ CC3^+^ cells per mm^2^ (*n* = 4 for each group). **(D)** Graph shows the number of CC1^+^ Olig2^+^ cells per mm^2^ (*n* = 4 for each group). **(E)** Graph showing the gram of grip strength (vehicle: *n* = 3, OA/LA: *n* = 4). *P < 0.05 assessed by two-sided Student’s *t*-test for panel **(C)** and two-way ANOVA followed by Sidak’s multiple comparison test for panel **(E)**. Scale bars: 25 μm for panels **(A,B)**.

We then investigated the *in vivo* role of OA/LA in oligodendrocyte survival during ALS progression. Immunohistochemical analysis revealed the expression of FFAR1 in the CC1^+^ Olig2^+^ OLs of SOD1^*G*93*A*^ mice (Figure 4A). SOD1^*G*93*A*^ mice were intraperitoneally administered an OA/LA cocktail from the pre-symptomatic (P60) to symptomatic (P100) stages (Avila-Martin et al., 2011). Immunohistochemical analysis showed a decrease in the number of CC3^+^ apoptotic oligodendrocytes in the anterior column of the OA/LA-treated SOD1^*G*93*A*^ spinal cord compared with that of vehicle-treated controls at the terminal point (Figures 4B, C), without changes in the number of CC1^+^ Olig2^+^ OLs (Figure 4D). These results suggest that circulating OA/LA prevents OL death during ALS pathogenesis. To investigate the functional significance of OA/LA treatment, we assessed the motor function of OA/LA-treated SOD1 mice by measuring grip strength. It was found that OA/LA treatment exerted a protective effect on loss of grip strength (Figure 4E), suggesting that the protective effect of OA/LA on oligodendrocyte also contributed to mitigating the ALS pathology.

### 3.4. OA and LA support motor neuron survival in SOD1^*G*93*A*^

**FIGURE 5:**
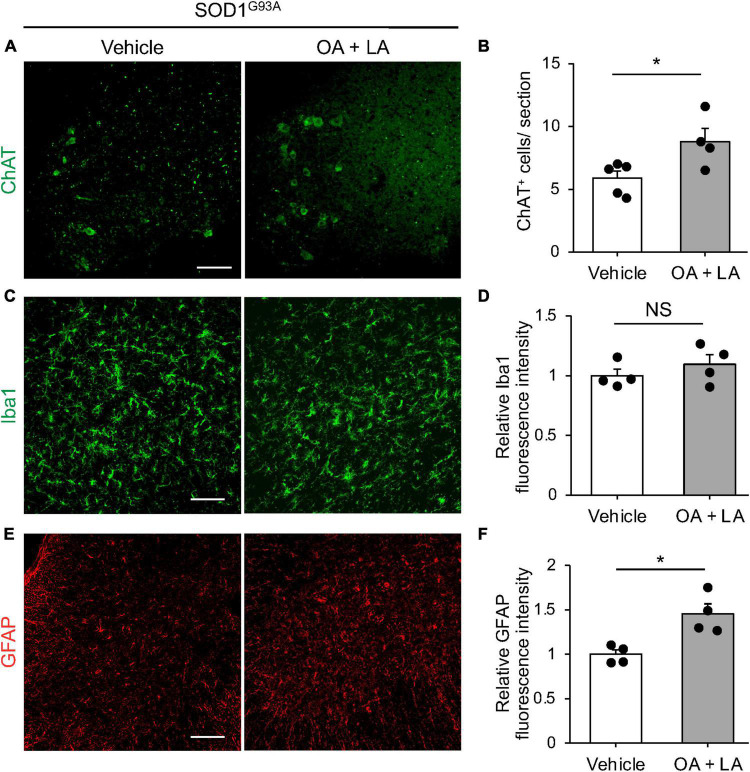
OA and LA prevent the degeneration of MNs in SOD1^G93A^ mice. **(A)** Representative images show the immunohistochemistry for ChAT in the spinal cord section. **(B)** The graph shows the number of ChAT^+^ cells per section (vehicle: *n* = 5, OA + LA: *n* = 4). **(C–F)** Representative images show the immunohistochemistry for Iba1 (green, **C**) and GFAP (red, **E**) in the spinal cord section. Graphs show the relative fluorescence intensity of Iba1 **(D)** and GFAP **(F)**, respectively (*n* = 4 for each group). Error bars represent mean ± SEM, *P < 0.05 assessed by two-sided Student’s *t*-test. NS, not significant. Scale bars: 100 μm **(A,C,E)**.

Finally, we examined whether circulating OA/LA affects other aspects of ALS pathology, such as MN loss (Rosen et al., 1993) and gliosis (Philips and Rothstein, 2014). Immunohistochemical analysis revealed a slight increase in the number of surviving ChAT^+^ MNs in the anterior column of the OA/LA-treated SOD1^*G*93*A*^ spinal cord compared to that in vehicle-treated SOD1 animals (Figures 5A, B). Regarding gliosis, the expression level of Iba1 (microglial marker) in the OA/LA-treated SOD1^*G*93*A*^ was comparable to that of vehicle-treated controls (Figures 5C, D). The expression level of GFAP (astroglial marker) was increased in the OA/LA-treated SOD1^*G*93*A*^ compared to that of vehicle-treated controls (Figures 5E, F), confirming the successful treatment of OA/LA *in vivo.* Taken together, these results suggest that OA/LA also have a protective effect on MNs.

## 4. Discussion

In this study, we applied global lipidomic analyses to identify circulating lipids that mediate ALS pathogenesis. We identified a robust decrease in circulating FFAs, including OA/LA, even in pre-symptomatic SOD1^*G*93*A*^ mice. OA/LA inhibited excitotoxic oligodendrocyte cell death via the cell surface receptor FFAR1. We also observed that the systemic administration of OA/LA ameliorated the loss of OLs and MNs in SOD1^*G*93*A*^ mice.

Alteration in lipid components has been reported in the cerebrospinal fluids and plasma in patients with ALS (Sol et al., 2021). Previous clinical studies have suggested interactions of FFAs with ALS; the levels of polyunsaturated fatty acids, including LA, was low in the FFA fraction of the blood and cerebrospinal fluids of patients with ALS (Henriques et al., 2015; Nagase et al., 2016), which is consistent with our rodent study. In ALS patients, functional decline correlates with increased resting energy expenditure, leading to a decrease in fat mass (Jésus et al., 2018), which might also decrease FFA release (Mittendorfer et al., 2009). In addition, high intake of polyunsaturated fatty acids is associated with a reduced risk of ALS (Fitzgerald et al., 2014). These reports support the notion that polyunsaturated fatty acids, including OA and LA, are protective, and reduction of polyunsaturated fatty acids in the plasma may be detrimental for ALS pathogenesis. Future studies should investigate the role of polyunsaturated fatty acids in plasma in human ALS pathology. Circulating FFAs are derived from stored triglycerides (TGs), which are the ester forms of FAs synthesized primarily in the liver and adipose tissue (Van Harmelen et al., 1999; Zechner et al., 2005). The concentration of postprandial circulating TGs was decreased in ALS mice due to increased lipid uptake in peripheral organs (Dupuis et al., 2004; Fergani et al., 2007). Furthermore, increased mobilization of lipids in ALS mice purportedly occurs to sustain metabolic requirements in peripheral glycolytic skeletal muscle, which has higher demands for fatty acid oxidization rather than as an energy source (Steyn et al., 2020). Thus, such changes in the metabolic demand for lipids might lead to a decrease in circulating FFAs prior to the onset of ALS. In contrast, we observed a tendency for circulating FFAs levels to increase as ALS progressed. This may be due to accumulated oxidative stress with disease progression, causing hydrolysis of tissue and membrane lipids to FFAs and release into the circulation (Barber et al., 2006; Nagase et al., 2016). Regarding other factors regulating circulating lipids, the gut microbiome is known to produce short-chain fatty acids (SCFAs, having fewer than six carbons) as metabolites of dietary fibers, which are released into the circulation (Dalile et al., 2019). SOD1^*G*93*A*^ mice exhibited changes in the composition and function of the gut microbiome prior to disease onset (Blacher et al., 2019). Although we could not detect SCFAs in our analysis, changes in the gut microbiome in ALS may also affect the systemic lipidome. Further investigations in peripheral organs are needed to elucidate the mechanism by which circulating FFAs levels decrease.

Our *in vitro* experiments revealed that OA/LA inhibit excitotoxic OL death. We treated OL with OA/LA at 30 μM, which is lower than plasma concentration (OA: 0.03–3.2 mM, LA: 0.2–5 mM) (Abdelmagid et al., 2015). However, FFAs, including OA and LA, were reported to be effective in cell death inhibition at a concentration of 10–50 μM *in vitro* (Ahn et al., 2013; Lee et al., 2022). Therefore, we used OA/LA at 30 μM to assess the cell death suppressive effects of OA/LA in this study. OA/LA suppressed glutamate-induced OL death via interaction with FFAR1, a G protein-coupled receptor. FFAR1 is coupled with G_q_ protein and activated by medium-chain fatty acids (with 6–12 carbons) and long-chain fatty acids (with more than 12 carbons), including OA and LA (Kasai et al., 2011; Ito et al., 2021). FFAR1 activation has been reported to trigger several downstream signaling cascades, including phosphatidylinositol-3 kinase (PI3K)/AKT, mitogen-activated protein kinase (MAPK)/extracellular signal-regulated kinase (ERK) signaling (Mena et al., 2016), which would protect OLs from excitotoxic apoptosis (Subramaniam and Unsicker, 2010). According to the contribution of other receptors, Gpr120 (also known as FFAR4) mediates the anti-apoptotic effect of FFAs (Katsuma et al., 2005). *Gpr120* is expressed in oligodendrocytes (Piatek et al., 2022); however, transfection of *Ffar4* siRNA did not influence the anti-apoptotic effect of OA/LA on OLs. Therefore, the anti-apoptotic role of FFAR4 might be differently regulated in cell types depending on the downstream signaling molecules they express. Further investigation is needed to elucidate the mechanism regulating the protection of OLs by OA/LA.

Our *in vivo* experiments revealed that systemic OA/LA administration ameliorated OL apoptosis without changes in the total number of mature OLs. The enhanced oligodendrogenesis and OL degeneration have been observed in pre-symptomatic SOD1^G93A^ mice (Kang et al., 2013), and the number of mature OLs is maintained during disease progression (Philips et al., 2013). These reports suggest that the oligodendrocyte turnover is enhanced in SOD1 mice, which might mask the changes in mature OLs with inhibition of apoptosis. In contrast, it has been reported that inhibition of oligodendrocyte apoptosis delayed disease progression and prolonged survival of SOD1 mutant mice (Kang et al., 2013) raising the possibility that inhibition of oligodendrocyte apoptosis is effective for ALS pathology, despite the low number of apoptotic oligodendrocytes even in pathogenic condition. As oligodendrocytes support motor neuron survival, suppressing oligodendrocyte apoptosis by OA/LA would be an effective treatment for ALS pathology. Indeed, OA/LA treatment ameliorated MN loss and motor dysfunction in SOD1 mice. Although we detected FFAR1 expression in OLs of ALS mouse spinal cords, FFAR1 expression was also observed in MNs of primate spinal cords (Ma et al., 2007). Thus, OA/LA may have a protective effect on MNs. We also observed enhanced astrogliosis in OA/LA-treated SOD mice. Astrocytes are reported to express fatty acid receptors or transporters, such as FABP7 and PPARγ, which regulate astrocyte reactivity, neuronal morphology, and metabolism (Fernandez et al., 2017; Hara et al., 2020). Further investigation is needed to elucidate the prospect of astrocyte-mediated oligodendrocyte cell death suppression by OA/LA.

Recent advantages of lipidomic analysis have revealed global changes in lipids in neurological diseases, such as Parkinson’s disease (Galper et al., 2022), Alzheimer’s disease (Proitsi et al., 2017), and multiple sclerosis (Gonzalo et al., 2012) in addition to ALS. Further evaluation of the multiple roles of FFA in the pathogenesis of CNS diseases, including ALS, may contribute to unveiling novel molecules that can serve as both biomarkers and therapeutic targets for CNS pathologies with abnormal lipid metabolism.

## Data availability statement

The raw data supporting the conclusions of this article will be made available by the authors, without undue reservation.

## Ethics statement

The animal study was reviewed and approved by the Committee on the Ethics of Animal Experiments of the National Institutes of Neuroscience, National Center of Neurology and Psychiatry (2021013R2).

## Author contributions

TM and ST performed the experiments. AU supported the drafting manuscript. TS advised the experiments. RM wrote the manuscript and supervised the project. All authors contributed to the article and approved the submitted version.
